# Contaminations, Sources, and Health Risks of Trace Metal(loid)s in Street Dust of a Small City Impacted by Artisanal Zn Smelting Activities

**DOI:** 10.3390/ijerph14090961

**Published:** 2017-08-25

**Authors:** Tingting Wu, Xiangyang Bi, Zhonggen Li, Guangyi Sun, Xinbin Feng, Lihai Shang, Hua Zhang, Tianrong He, Ji Chen

**Affiliations:** 1 Laboratory of Karst Environmental and Geological Disaster Prevention and Control, Ministry of Land and Resources, Guizhou University, Guiyang 550003, China; wutingtingcc@163.com (T.W.); hetianrong@139.com (T.H.); 2 State Key Laboratory of Environmental Geochemistry, Institute of Geochemistry, Chinese Academy of Sciences, Guiyang 550081, China; sunguangyi@mail.gyig.ac.cn (G.S.); fengxinbin@mail.gyig.ac.cn (X.F.); shanglihai@mail.gyig.ac.cn (L.S.); zhanghua@mail.gyig.ac.cn (H.Z.); jigerchen@163.com (J.C.); 3 State Key Laboratory of Biogeology and Environmental Geology, School of Earth Science, China University of Geosciences, Wuhan 430074, China; bixy@cug.edu.cn; 4 Guizhou Provincial Laboratory for Mountainous Environment, Guizhou Normal University, Guiyang 550001, China

**Keywords:** Zn smelting activities, heavy metal(loid), street dust, contamination assessment, health risk assessment

## Abstract

To investigate the impact of artisanal zinc smelting activities (AZSA) on the distribution and enrichment of trace metal(loid)s in street dust of a small city in Guizhou province, SW China, street dust samples were collected and analyzed for 10 trace metal(loid)s (Cr, Co, Ni, Cu, Zn, As, Cd, Sb, Pb, and Hg). Meanwhile, the health risks of local resident exposed to street dust were assessed. The result showed that the average concentrations of 10 elements were Zn (1039 mg kg^−1^), Pb (423 mg kg^−1^), Cr (119 mg kg^−1^), Cu (99 mg kg^−1^), As (55 mg kg^−1^), Ni (39 mg kg^−1^), Co (18 mg kg^−1^), Sb (7.6 mg kg^−1^), Cd (2.6 mg kg^−1^), and Hg (0.22 mg kg^−1^). Except Ni, Co, and Cr, other elements in street dust were obviously elevated compared to the provincial soil background. Pb, Zn, Cd, Sb, and Cu were at heavy to moderate contamination status, especially Pb and Zn, with maximums of 1723 and 708 mg kg^−1^, respectively; As and Hg were slightly contaminated; while Cr, Ni, and Co were at un-contaminated levels. Multivariate statistical analysis revealed AZSA contributed to the increase of Pb, Zn, Cd, Sb, As, and Hg, while, natural sources introduced Ni, Co, Cr, and Cu. The health risk assessment disclosed that children had higher non-carcinogenic risk than those found in adults, and As has hazardous index (HI) higher than 1 both for children and adults, while Pb and Cr only had HIs higher than 1 for children, other elements were relatively safe. For carcinogenic risks, the major concern was As, then a lesser concern for Cr. The study showed that although the scale of AZSA was small, the contamination of heavy metal(loid)s in street dust and associated health risks were severe.

## 1. Introduction

Street dust is comprised of solid particles with complex compositions that is deposited on outdoor surfaces and can be easily re-suspended into air by wind [1]. It originates either from natural sources (e.g., re-suspension of soil and weathered materials), or various anthropogenic activities (e.g., vehicular traffic, industrial plants, power generation facilities, residential fossil-fuel burning, construction and demolition activities, etc.) [2]. Street dust has high surface area and is easily transported and deposited [3], so it acts as both the ‘source’ and ‘sink’ of different kinds of pollutants in the city environment, including trace metal(loid)s.

Potentially harmful metal(loid)s are important contaminants in street dust in urban areas, which not only reflect the characteristics of industries and the environmental qualities, but also affect public health by disturbing the central nervous, blood-forming, cardiovascular, renal, and reproductive systems [4,5]. Toxic metal(loid)s in street dusts may enter the human body mainly via three routes: direct inhalation, ingestion, and dermal contact absorption [6]. In particular, children are more vulnerable than adults because of their frequent hand-to-mouth activities, higher absorption rate of the digestion system, and hemoglobin sensitivity to toxic metal(loid)s [7].

Metal smelting is one of the primary anthropogenic sources of heavy metal(loid)s to the environment [8,9]. During the high temperature smelting process, heavy metal(loid)s in ores are either evaporated into the flue gas or remain in their solid residue. If proper pollution control technologies are not applied, these heavy metal(loid)s in mineral ores would eventually escape into the surrounding environment [9,10]. Zinc is an important nonferrous metal that is used in galvanizing, brass, bronze, zinc based alloys, chemicals, and dry batteries. China is the largest zinc producer in the world since 1991 and produced 44% of the world total production in 2015 [11]. There are two types of technique for Zn production, one is the hydrometallurgical process; the other is the pyrometallurgical process, which consists of four sub-types: retort Zn production, the imperial smelting process, electric furnace process, and artisanal zinc smelting process [12,13]. Artisanal zinc smelting is an easy, small scale, and outdated zinc smelting technique, which extracts Zn from Zn concentrate with burning coal as heat source and reducing agent [9]. This method has been very popular in many provinces in southern China—such as Guizhou, Yunnan, Sichuan, and Hunan—in the last several centuries [14]. Since no pollution control measures were used, this kind of activity released a huge amount of acid gas (SO_2_) and a range of toxic metals into the environment [10,12].

Although, there were plenty of studies on the emissions of trace metal(loid)s from artisanal zinc smelting, and metal(loid)s contamination in different environmental compartments (water, soil, sediment) and foodstuff in the artisanal zinc smelting area in China [10,15], no attention had been paid to the ground dust or street dust. So, in order to have a comprehensive understanding of trace metal(loid)s contamination, this study investigated the contamination of trace metal(loid)s in the street dust of a small city engaged in artisanal zinc smelting in Guizhou, China. At the same time, the health risk due to exposure to street dust was also evaluated.

## 2. Materials and Methods

### 2.1. Study Area

**Figure 1:**
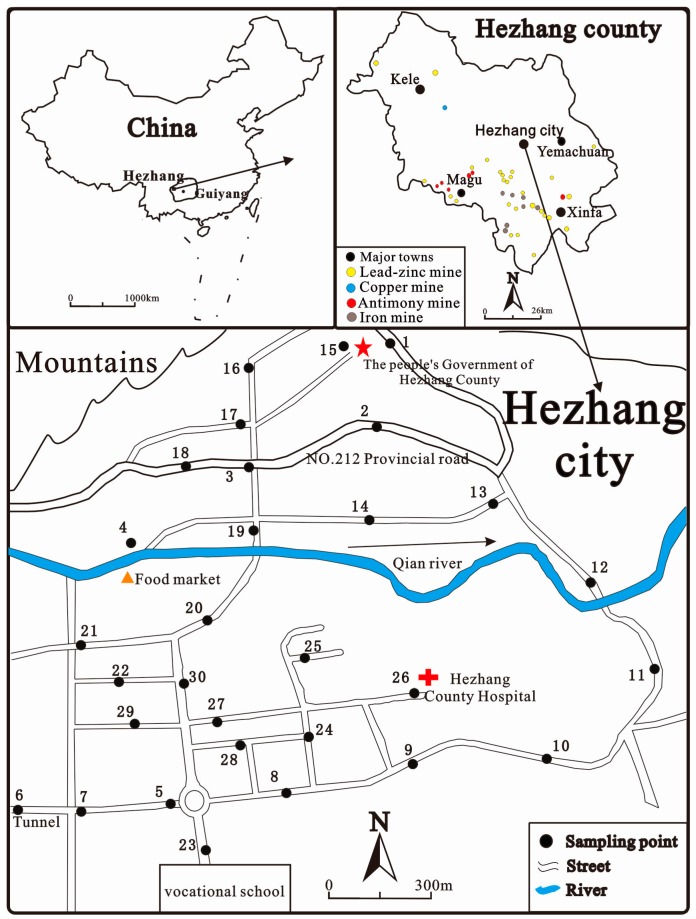
Sketch map showing the study area and the street dust sampling sites.

The study area is Hezhang city, which lies in the northwest Guizhou province (Figure 1), southwestern China. The distance from Hezhang city to the capital of Guizhou province, Guiyang, is about 300 km. Hezhang city locates at a plateau mountainous area (Yunnan-Guizhou Plateau) with an average altitude of 1710 m above sea level. The average annual temperature and precipitation are 13.4 °C and 854 mm, respectively. The study area belongs to the subtropical monsoon climate regions, with prevailing wind from the southwest in summer and northeast in winter. The urban residential population in Hezhang city was 18,000 in 2009 when this study was carried out, and the construction area of the city was about 3 km^2^. The city is located in a valley, the Qian river flows through the city from the west to the east, both north and south sides of the city are surrounded by mountains with elevation up to 2300 m above sea level.

Hezhang city is the capital of Hezhang county, the city has an abundance of lead, zinc, copper, iron, and coal resources, as shown in Figure 1. Lead and zinc ore reserves in Hezhang county ranked No. 1 in Guizhou, with around 8 million tons [16]. Since the area hosts both coal and zinc ores, this area has the longest zinc smelting history in China, dating back up to 1000 years ago [14]. After the Chinese reform and opening up in the early 1980s, zinc smelting in Hezhang county developed rapidly with the slogan of “development of township enterprises”. Artisanal zinc smelting was encouraged and a substantial increase in crude zinc production was achieved in the 1990s. In 2000, Zn production in this county reached to a historical level at 110,300 tons. The main areas of artisanal zinc smelting in Hezhang county were concentrated in Magu town and Yemachuan town—36 km and 17 km to Hezhang city, respectively—as shown in Figure 1. In addition, there were a few artisanal zinc smelting stoves sporadically distributed in the valleys around Hezhang city. Due to the absence of any environmental protection measures during the artisanal zinc smelting process, metal atmospheric emission levels—such as Hg and Cd—were severely high [15] and the local soil, sediment, air, food, and moss were extremely contaminated by Pb, Zn, and Cd [10,17]. With awareness of environmental protection, the local government started to prohibit artisanal zinc smelting activities since the early 21st century, and completely banned this activity in 2006.

### 2.2. Sample Collection

All the field sampling was conducted in September 2009, three years after the completed banning of artisanal zinc smelting. A total of 30 street dust samples were collected throughout the Hezhang city in 13 September 2009 (Figure 1), equivalent to 10 sampling sites per square kilometer. As a control, three samples of street dusts were collected from the Huaxi district in a southern suburb of Guiyang where no industries exist and with a distance of about 18 km to the city center. The data in Guiyang city from the literature was also used as a reference. During the field work, the weather was stable, calm, and cloudless. At each site, around 200–500 g of street dust was obtained from the road surface or the sidewalks by using a plastic brush and dustpan within an area about 5–20 m^2^, then the samples were transferred into air-tight polyethylene bags for storage. At the same time, to disclose the possible sources of trace metals and metalloids in street dusts of Hezhang city, 23 composite surface soil samples (0–20 cm) were taken from maize fields at a range of 3 to 10 km from the city. Agricultural soils may also be impacted by the artisanal Zn smelting activities and fertilization, to exclude these influences, we also collected six grass land soils 25 km away the city. Additionally, three limestone samples in Guizhou were taken to reflect the chemical compositions of construction material, such as cement. All the samples were air-dried in the laboratory, and the coarse impurities—such as stone, cigarette butts, plastic, and residues of plant—were removed, then the rest were ground with an agate mortar and pestle to pass through a 100-mesh nylon sieve (0.149 mm) and thoroughly homogenized in the lab.

### 2.3. Sample Analysis

Ten trace metals and metalloids in all samples were determined, including antimony (Sb), arsenic (As), cadmium (Cd), cobalt (Co), chromium (Cr), copper (Cu), lead (Pb), mercury (Hg), nickel (Ni), and zinc (Zn). Concerning the chemical analysis of Hg and As, 0.2–0.5 g of sample was digested in a 95 °C water bath for 1 h using a mixture of 5 mL aqua regia (HCl (40%, *v/v*) and HNO_3_ (65%, *v/v*) at 3:1 *v/v*) and 5 mL Milli-Q water [9]. An appropriate aliquot of the digest was analyzed using cold vapor atomic fluorescence spectrophotometer (for Hg, Tekran Model 2500, Tekran Instruments Corp., Toronto, ON, Canada; for As, AFS-920, Beijing Jitian Instrument Corp., Beijing, China). For the analysis of the remaining eight metal(loid)s, a wet digestion procedure coupled with detection with inductively coupled plasma-mass spectrometry (ICP-MS, ELAN DRC-e, PerkinElmer Inc., Fremont, CA, USA) was adopted from Qi and Grégoire [18]. Briefly, 50 mg of sample were digested using 1 mL of HF (40%, *v/v*) and 1 mL of HNO_3_ (65%, *v/v*) in the PTFE-lined stainless steel bombs heated to 190 °C for 24 h. Insoluble residues were dissolved using 6 mL of 65% *v/v* HNO_3_ heated to 140 °C for 5 h. After cooling down, about 0.4 mL of the digest was transferred to a centrifuge tube, and added with 500 ng rhodium in a liquid solution, then made up to approximately 10 mL with Milli-Q water. Rhodium was used as an internal standard to correct for matrix effects and instrumental drift. For quality assurance and quality control (QA/QC), the method blanks, duplicates and standard reference materials (GSS-5 and GSD-5, representing soil and sediment, respectively) were analyzed. The mean recoveries for 10 elements (C (element, measured)/C (element, certified) × 100) in the two SRMs were between 87% and 110%. The duplicate samples showed that the bias was less than 5%, indicating the samples are thoroughly homogenized.

In addition, pH in samples was determined in a 2.5:1 (*w/w*) water/dust suspension using a pH meter, and organic material (OM, %) content was determined by potassium dichromate method [19].

### 2.4. Contamination Assessment

Geo-accumulation index (*I_geo_*) approach was used to assess the contamination status for a single element in street dust [20]. This method is a kind of quantitative index to study the degree of trace metal(loid) pollution in soil or sediment. In recent years, many researchers have widely applied this method to the assessment of trace metal(loid) pollution in urban street dust [2,21]. The method calculated *I*_geo_ using the following equation:

(1) $$I_{geo}=\log_{2}\left[\frac{C_{i\mathrm{Sample}}}{1.5\times C_{i\mathrm{Background}}}\right]$$

where *I_geo_* is the index of geo-accumulation of *i* element; *C_i_*_Sample_ is the measured concentration of *i* metal(loid) in sample; and *C_i_*_Background_ is the geochemical background value of *i* heavy metal(loid) in soils. In this study, the background of trace metal(loid)s in the surface soil layer (0–20 cm) in Guizhou province [22] was taken as the reference value; coefficient of 1.5 was introduced in this equation to minimize the effect of possible variations in the background values. This method assigns the metal/metalloid pollution to seven (0–6 grade) enrichment classes, ranging from background concentration to very heavily polluted. According to Müller [20], the *I*_geo_ for each element is calculated and classified as: uncontaminated (*I_geo_* ≤ 0); uncontaminated to moderately contaminated (0 < *I_geo_* ≤ 1); moderately contaminated (1 < *I_geo_* ≤ 2); moderately to heavily contaminated (2 < *I_geo_* ≤ 3); heavily contaminated (3 < *I_geo_* ≤ 4); heavily to extremely contaminated (4 < *I_geo_* ≤ 5); and extremely contaminated (*I_geo_* ≥ 5).

### 2.5. Health Risk Assessment

In this study, the health risk of human exposure to street dust of Hezhang city was assessed using method adopted from the U.S. EPA [23,24]. Both the carcinogenic and non-carcinogenic effects were evaluated. The health risk assessment included estimation of the amount of pollutants entering the body and the relationship between the dose and the negative health effects. Among the 10 studied elements, five (As, Cr, Ni, Cd, Co) are regarded as carcinogenic factors according to the U.S. Agency for Toxic Substances and Disease Registry (ATSDR), while the other five heavy metals (Cu, Zn, Sb, Pb and Hg) are non-carcinogenic substance, but they could cause chronic poisoning. In this study, all the elements were used as total concentration in the street dust. Non-carcinogenic hazard was assessed using hazard index (HI), the HI considered three main pathways street dust entering the residents body, namely: (1) direct ingestion through the hand-moth way ($D_{ing})$; (2) inhalation of re-suspended particles through the mouth and nose ($D_{inh}$); and (3) dermal absorption of trace elements in particles adhered to exposed skin ($D_{dermal}$). The dose received through each of the three pathways was calculated using Equations (2)–(4), which were adopted from U.S.EPA (1989, 1996) [23,25] and Zheng et al. [7], the parameters and values in these Equations (2)–(4) were shown in Table 1. Hazard quotient (HQ) for non-carcinogenic effects was applied to each exposure pathway in the analysis, as shown in Equation (5). The approach assumes that simultaneous sub-threshold exposures to toxic metal(loid)s could result in adverse health effects and the magnitude of the adverse effect will be proportional to the sum of the ratios of the sub-threshold exposures to acceptable exposures [23], where RfD is the reference dose (mg kg^−1^ day^−1^) and D*_i_* is the daily exposure amount of the selected metal(loid) (mg kg^−1^ day^−1^) through the *i*th pathway. Hazard index (HI) in Equation (6) is equal to the sum of HQ_i_. If the value of HI is less than one, it is assumed that there is no significant risk of non-carcinogenic effects. If the HI exceeds one, then there is a chance that non-carcinogenic effects occur, with a probability which tends to increase as the value of the HI increases [26].

(2) $$D_{ing}=C\times \frac{IngR\times EF\times ED}{AT\times BW}\times 10^{-6}$$

(3) $$D_{inh}=C\times \frac{InhR\times EF\times ED}{AT\times BW\times PEF}$$

(4) $$D_{dermal}=C\times \frac{EF\times ED\times SA\times AF\times ABSd}{AT\times BW}\times 10^{-6}$$

(5) $${\mathrm{Hazard}\;\mathrm{quotient}\;\mathrm{of}\;i}^{th}\;\mathrm{pathway}\;\left(HQ_{i}\right)=D_{i}/\mathrm{Rf}D_{i}$$

(6) $$\mathrm{HI}=\sum HQ_{i}$$

For carcinogens, the lifetime average daily dose (LADD) of the *i*th pathway was estimated in Equation (7), then the carcinogenic risks—which is the probability of developing any type of cancer in the whole life of an individual due to exposure to carcinogenic hazards—was calculated using Equation (8), where SF is the slope factor for carcinogenicity per unit exposure level (mg kg^−^^1^ day^−^^1^). If carcinogenic risk <1 × 10^−^^6^, the carcinogenic risk is negligible; if carcinogenic risk >1 × 10^−^^4^, the risk of developing cancer is high; and if carcinogenic risk values remain within the range of 1 × 10^−^^6^ and 1 × 10^−^^4^, it is an acceptable or tolerable risk to social stability and human health [29].

(7) $$LADD_{ing}=\frac{C\times EF}{AT\times PEF}\times \left(\frac{Ing_{child}\times ED_{child}}{BW_{child}}\times \frac{Ing_{adult}\times ED_{adult}}{BW_{adult}}\right)$$

(8) $$\mathrm{Carcinogenic}\;\mathrm{Risk}=\sum^{\text{​}}LADDi\times SFi$$

In Table 1, C (exposure-point concentration, mg kg^−1^) used in Equations (2)–(7), is the upper limit of the 95% confidence interval of the mean (95% UCL), that is considered to yield an estimate of the “reasonable maximum exposure” (U.S. EPA, 1989) and is calculated using statistical software SPSS 15.0 (SPSS Inc., Chicago, IL, USA) for Windows. Table 2 summarized the 95% UCL of studied trace metal(loid)s, the reference dose (RfD), and slope factor (SF) for each element that based on U.S. EPA (1996, 2002), Keshavarzi et al. (2015), Wan et al. (2016), Ferreira-Baptista and Miguel (2005) [25,27,30,31,32].

### 2.6. Data Processing Method

The obtained data were statistically analyzed using SPSS 15.0, also the Pearson’s correlation coefficient and the Principal Component Analysis (PCA) were performed using SPSS tool. The data was graphed with Origin 8.0 (Origin Lab, Northampton, MA, USA).

## 3. Results and Discussion

### 3.1. Trace Metal(loid)s in Street Dust

The descriptive statistics of studied metal(loid)s, as well as OM, pH of street dust in Hezhang city were presented in Table 3. The average concentration (median) of 10 elements was decreased in the following sequence, Zn (1039 mg kg^−1^), Pb (423 mg kg^−1^), Cr (119 mg kg^−1^), Cu (99 mg kg^−1^), As (55 mg kg^−1^), Ni (39 mg kg^−1^), Co (18 mg kg^−1^), Sb (7.6 mg kg^−1^), Cd (2.6 mg kg^−1^), and Hg (0.22 mg kg^−1^). Zn and Pb were the most abundant elements, with maximums of 1723 and 708 mg kg^−1^, respectively. pH and OM averaged at 8.5% and 4.0%, respectively, reflecting an alkaline and rich organic matter property.

Compared to the reference Guizhou soil (Table 3) [22], all metals—except Co, Ni, and Cr—in street dust were much elevated. Pb and Zn were 12.0 and 10.4 times higher than the provincial soil background. Cd, Sb, Cu, As, and Hg were elevated 2–4 times of the soil background. Additionally, the averages of Pb, Cd, Zn, Sb, and As in street dust of Hezhang city were 8.9, 7.9, 6.6, 3.3, and 2.2 times higher than that of the control sites in Huaxi district of Guiyang city, respectively, revealing that the impact of Zn smelting on the enrichment of these metal(loid)s is severe. As an important material source of street dust, local agricultural soils contained around twice as much Cr, Co, and Ni than the street dust in Hezhang city (Table 3), while, Pb, Zn, As, and Sb levels in local agricultural soils were much lower than in the street dust. Although some agricultural fields also suffered contamination from artisanal zinc smelting activities, street dust showed more severe contamination statistically, this was similar to other cities with zinc smelting [9], the main reason for this phenomenon was the dilution effect occurring at the plough layer of agricultural soils, while the street surface was impermeable which magnified the contamination levels. Pb, Zn, Cd, Hg, As, and Sb in street dust were even further elevated than in the regional grass land soil, reflecting the obvious impact of zinc smelting activities. On a global scale (Table 4), trace metal(loid)s in street dust of Hezhang city were generally identical to that of Zhuzhou [9] and Huludao [7] which are both affected by large scale Zn smelting activities in China, but also obviously lower than Avilés in Spain which is also affected by large scale Zn smelting activities [33]. For these cities, Zn, Pb, As, Cd, and Sb were not notably higher than that non-zinc smelting cities, and the varying concentrations of Zn, Pb, As, Cd, and Sb in these zinc smelting cities reflected the difference in smelting scale and the pollution control measures that were applied. Although the Zn smelting scale in Hezhang was much smaller than Zhuzhou and Huludao in China, the contamination status was comparable, indicating higher pollution intensity from the artisanal zinc smelting method. For non-zinc-smelting cities, including some cities affected by coal mining, such as Huainan in China, the vast majority of elements demonstrated generally low level presence [34]. Nevertheless, we found some elements—like Zn, Cu, Sb, and Cr—in a range of megacities like Hongkong, Beijing, Shanghai, Suzhou, Birmingham, and Tehran, were at relatively higher levels, this might reflect vehicle emissions, either from vehicle tailpipe exhaust emissions or non-exhaust emissions (such as component abrasion). However, the non-exhaust emissions would account for more than 90% of the total emissions from road traffic after the late 2000s [35], since these elements were widely used in a lot of wearable parts of cars, such as brake linings, clutch plates, tires, etc. [36].

The zinc smelting processes not only discharged trace metals, but also emitted acidic compounds such as sulfur dioxide. However, urban street dust displayed significantly higher alkalinity (pH = 8.5) than the agricultural soil (pH = 5.9 and 6.2 in local and Guizhou soils, respectively), that could be ascribed to inclusion of a high proportion of particulate derived from construction materials such as concrete and limestone. The tendency of higher OM content in the street dust (5.0%) possibly reflects practices within urban areas concerning the handling of organic refuse and waste (e.g., garbage, offal, and litter) [9].

The homogeneity of studied elements in Hezhang city could be reflected by the skewness and coefficient of variation (CV). As shown in Table 3, the skewness of four metals (Zn, As, Sb, and Pb) was approximately zero (<0.4), reflecting uniform distribution, i.e., the difference between different sites, are negligible and these elements were homogenously scattered in the whole city. For the other six metals (Cd, Cr, Cu, Hg, Ni, and Co), the skewness was greater than zero (0.8-4.9), indicating the distributions were somewhat positively skewed, with mean values at the right side of the peak. Among them, Cu, Cr, and Hg had relatively higher skewnesses of 4.9, 4.3, and 2.8, respectively, suggesting few relatively higher outlier data and the heterogeneity of these metals. The CV values revealed a similar result. It is described that CV ≤ 20% indicates low variation, 21% ≥ CV ≤ 50% indicates moderate variation, 50% ≥ CV ≤ 100% indicates high variation, and above 100% is exceptionally high variation [50]. Most studied metal(loid)s, pH, and OM in Table 3 showed low to moderate variation (7–44%), only Cr (57%) and Hg (57%) showed high variation and Cu (136%) showed exceptionally high variation. The highest Cr content appeared in the southwest part of the city, where sites 28 and 24 had concentrations as high as 508 and 204 mg/kg, respectively; higher Hg concentrations manifested in the north part of city (sites 16–18) with concentrations of 0.39-0.89 mg kg^−1^ at a site close to the hospital (site 26, Hg: 0.54 mg kg^−1^); while the southwest and central to east part of the city had higher Cu content. In the southwest, site 6 had the highest Cu concentration (1212 mg kg^−1^), then sites 28–30 (203–218 mg kg^−1^), in central to east part of the city, sites 12–14 also had relatively higher Cu (200–229 mg kg^−1^).

### 3.2. Contamination of Trace Metal(loid)s in Street Dust

**Figure 2:**
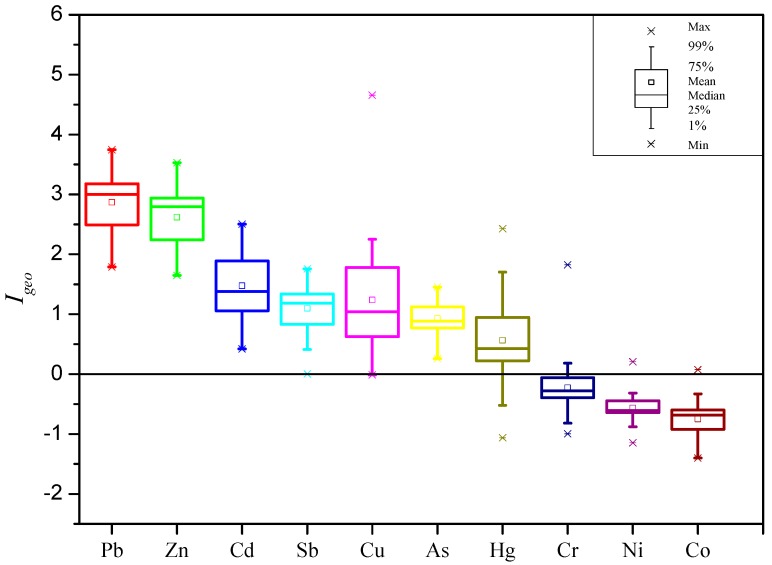
Box plot of geo-accumulation index value (*I_geo_*) of trace metal(loid)s in street dust of Hezhang city.

Taken the background of surface soil in Guizhou, shown in Table 3, as a reference, the contamination levels of trace metal(loid)s in street dust of Hezhang city were assessed by the geo-accumulation index method. The result showed the contamination levels decreased in the following order by median of *I_geo_*, Pb > Zn > Cd > Cu > Sb > As > Hg > Cr > Ni > Co (Figure 2). The most contaminating elements were Pb and Zn, with median *I_geo_* values of 3.00 and 2.80, respectively, reflecting moderate to heavy contamination levels. For Cd, Sb, and Cu, the median *I_geo_* were 1.39, 1.20, and 1.04, respectively, indicating moderate contamination. The average of *I_geo_* for As and Hg were 0.95 and 0.73, respectively, indicating a slight contamination. The remaining elements, Cr, Ni, and Co, have average *I_geo_* values less than 0, indicating no contamination.

### 3.3. Sources of Trace Metal(loid)s in Street Dust

**Figure 3:**
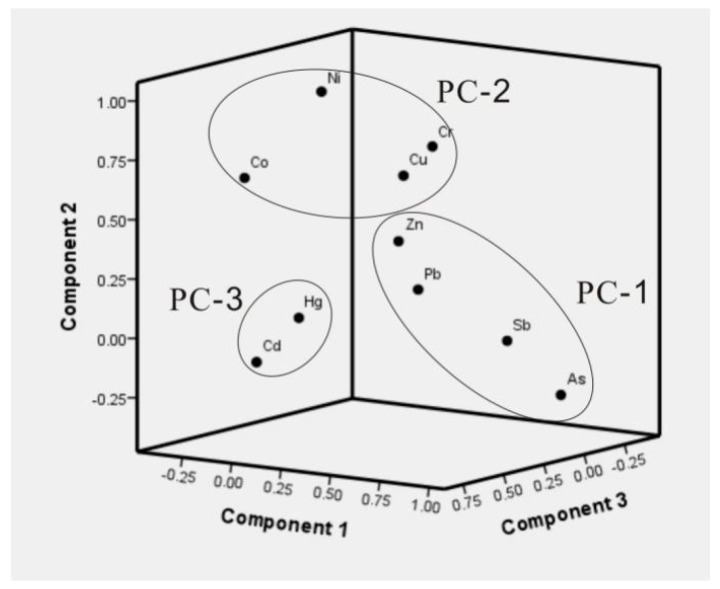
3D plot of scores for investigated elements obtained from PCA results for street dust of Hezhang city.

Trace metal(loid)s contamination may be caused by many sources. To determine the possible source of trace metal(loid)s in street dust of Hezhang city, the multivariate statistical analysis, including correlation analysis and principal component analysis (PCA), were performed. The result of correlation analysis in Table 5 indicated that Ni and Cr, Co, Cu; Zn and Sb, Pb; Sb and As, Pb were correlated at the 0.01 significant level. To a lesser degree, Zn and Cu, Cd, Hg, Ni; Pb and As, Cd, Hg were correlated at the 0.05 significant level. The close correlation between different elements suggested that they may share the common sources. To better disclose the possible sources, PCA analysis was conducted, the results were shown in Table 6. The principal components (PC) were extracted with eigenvalues higher than 1, the PC-1 explained 32.6% of total variance and mainly included As, Sb, Pb, and Zn; the PC-2 explained 24.2% of total variance with significant loadings of Ni, Cr, Cu, and Co. The PC-3 explained 12.0% of variance which was mainly contributed by Cd and Hg. These three principal components totally explained 68.8% variance of the data and were projected in a three-dimensional space, as shown in Figure 3. Considering the local human activities and geological background, it can be confirmed that PC-1 and PC-3 essentially represent emissions related to the Zn smelting activities, since Zn smelting activities emitted up to ten trace metals and metalloids into the surrounding environment—including Pb, Zn, Cd, Sb, Ag, In, Tl, Hg, As, Cu, Bi and Ga [9]—while the second principal component represents the natural sources. The artisinal Zn smelting processes emitted trace metal(loid)s into the local environment in Hezhang principally via two routes: smelting flue gas and abandoned smelting wastes [10], largely based on the metal(loid)’s boiling points. Hg and Cd in Zn ores would be evaporated and entered the gas-phase during the high temperature smelting process due to their low boiling points (357 °C for Hg and 765 °C for Cd, respectively), and they were depleted in the solid residues, while other metals and metalloids mainly remained in the waste residue [10]. PCA results indicated that elements in PC-1 that had higher boiling points were derived from the abandoned solid smelting wastes and elements in PC-3 with lower boiling points were from the smelting flue gas or fine particles. Zn smelting will release some Cu to the environment [9], while its contribution to street dust in Hezhang city was not obvious, as the score of Cu in PC-1 is not high (0.287). For Hg, some sites in Hezhang city may also affected by other human activities, such as the hospital that uses thermometers containing Hg. While Sb and Cu are components of brakes and can be also introduced by traffic, and As is present in coal used in the Zn smelting, and may be also another contribution to the local environment. However, compared with the Zn smelting activities, these sources’ importance would not be too high. West Guizhou province is located at the edge of Permian–Triassic Emeishan flood basalts [51]—which contain relatively higher contents of Cr, Ni, Co, Cu, Se, Sr, V, and Ti, and this has been verified by the local soil profiles [10] and the surface agricultural soils in this study, as shown in Table 3—so elements in PC-2 would largely originate from the natural source. For construction materials, such as limestone which is used to produce cement, contained negligible trace metals and metalloids, except Ni (Table 3), hence, the contribution from the construction materials would also be very small.

### 3.4. Health Risk of Trace Metal(loid)s in Street Dust

For non-carcinogenic effects of trace metal(loid)s on children and adults, the calculated overall risk and the corresponding contribution for each exposure pathway are summarized in Table 7. Ingestion of street dust particles appeared to be the main exposure pathway for As, Pb, Cr, Sb, Cd, Cu, Zn, Ni, Hg, and Co to children, followed by dermal contact and inhalation routes, respectively. For both children and adults, the rank for exposure risk among the three routes were decreased in the order of ingestion > dermal contact > inhalation, except for arsenic, which was in the order of dermal contact > ingestion > inhalation due to the higher dermal absorption factor for arsenic (0.03) than other elements (0.001). In general, children had higher exposure doses for all three pathways than those of adults. Namely, children had 2~5 times higher non-carcinogenic risk for all studied elements than that of adults, indicating that children are confronted by greater harmful health risks due to the street dust than adults. This was similar to other reports in Beijing, Nanjing, Shiraz, Urumqi, Huludao, and Luanda [7,21,30,31,32,52].

Hazard index (HI) decreased in the order of As > Pb > Cr > Sb > Cd > Cu > Zn > Ni > Hg > Co for children, and As > Cr > Pb > Sb > Cd > Cu > Zn > Ni ≈ Hg ≈ Co for adults. HIs of arsenic both for children (4.379) and adults (1.650) were higher than 1, indicating that non-carcinogenic effects may occur. HI of Pb and Cr for children, with 1.785 and 1.002, respectively, were also exceeding 1, showing their possible non-carcinogenic effects on children. While for other metal(loid)s, HIs both for children and adults were lower than the safety limit of unity (<1), indicating less non-carcinogenic risks. Concerning the two population groups, they both experience potential health risks from arsenic exposure due to street dust, since HI > 1. However, according to De Miguel et al. [53], adverse health effects may occur when HI values > 0.1 in the child cohort. Consequently, Sb in Hezhang with HI = 0.377 for children, the exposure to the street dust cannot be overlooked. Arsenic has strong toxicity, mainly afflicting the mucous membrane by directly damaging the capillaries; lead absorption causes direct damage to the human nervous system, a fetus may suffer congenital mental decline, the elderly may suffer dementia; chromium can reduce the biochemical process of oxygen demand, resulting in asphyxia; chromium salt aggravates the stomach; antimony will damage the skin, but can also afflict the bones, liver, and kidney [54].

The carcinogenic risks owing to exposure to As, Cd, Co, Cr, and Ni in street dust were shown in Table 8. The carcinogenic risk levels of Cd, Co, and Ni were within the order of magnitude of 10^−8^–10^−9^, which means that they pose a negligible risk. In terms of As, the calculated value of cancer risk, 4.90 × 10^−4^, exceeded the threshold values of 10^−6^ which is an internationally accepted precautionary risk criterion. Cr risk (3.06 × 10^−6^) also exceeded the criterion. Arsenic in street dust was introduced by the Zn smelting activities and Cr was naturally enriched in the street dust as mentioned above. As a carcinogen, arsenic can cause lung and skin cancer and Cr can trigger lung cancer and stomach cancer if an excessive buildup of both elements occurs [55]. The results reflected a possible of cancer risk due to As and Cr exposure through exposure to street dust in Hezhang city, while further research should be undertaken to reveal the synergistic influence from Cr and As on human health.

Comparing the non-carcinogenic health risk in the present study and different cities in the world (Table 9), the human health risk was significantly greater than Beijing, Nanjing, Luanda, and other places, while it was comparable to Huludao and Zhuzhou in China [7,9,21,31,32]. Beyond that, for children, the total exposure HIs of As, Pb, and Cr with value higher 10^−1^ appeared in several studies (seen in Table 9), especially As and Pb, these two elements could trigger neurological and developmental disorders [32]. For carcinogenic risk (Table 10), arsenic was the only elements for concern in most cities, and other elements (Cd, Cr, Co, and Ni) was at relatively safe level. For arsenic, the HI means of these cities are more than 0.1 and the cancer risk means are in 4.9 × 10^−4^ to 10^−8^ level. While the data indicated that HI and cancer risk from As in Hezhang (4.9 × 10^−4^) is much more serious than other cities (Zhuzhou, Beijing, Luanda, etc.), indicating a more worrying polluted status. Metalloid arsenic (As) is ranked number one in the list of priority pollutants harmful to human health by the ATSDR (2007) [56]. Exposure to As will result in the development problems as well as numerous other health disorders [57]. For chromium, only a few cities had HI higher than 0.1 and the cancer risk means are in 3.1 × 10^−6^ to 10^−8^ level. For lead, Hezhang had second higher HIs both for children and adults (1.8 and 0.3, respectively), only lower than Zhuzhou. This showed the potential health risks of lead in Hezhang. For adults, the total HIs from ingestion, dermal contact, and inhalation for Cr, Co, Ni, Cu, Zn, Cd, Sb, Pb, and Hg in these cities were lower than 1, indicating no potential health risk would occur.

## 4. Conclusions

Based on this study, we can draw the following conclusions: although the scale of artisanal Zn smelting in Hezhang was small, the heavy metal(loid)s contamination in street dust was similar to that of large-scale zinc smelting cities in the world. The main pollutants in street dust of Hezhang city were Pb and Zn. Six studied elements (Pb, Zn, Cd, As, Sb, and Hg) were mainly derived from the zinc smelting activities, while other four elements (Cr, Ni, Co, and Cu) were primarily originated from the natural source. Human health assessment showed that the health risk of children was greater, mainly through hand–mouth routes, the main factors affecting the human body were elements of As, Pb, Cr, and Sb. The findings are particularly significant when one considers the fact that the children in Hezhang city are the most sensitive subpopulation to potential harmful metal(loid)s exposure in street dust. Preventing intake by ingestion of street dust will effectively reduce carcinogenic and non-carcinogenic risks of potentially harmful metals and metalloids for children. Since the abandoned artisinal Zn smelting solid wastes were the main source of harmful trace metal(loid)s to the local environment, proper treatment to obstruct the contact of these solid wastes from the biosphere would be the first priority after cessation of artisanal Zn smelting activities.

## Figures and Tables

**Table 1:** Values and factors used for non-carcinogenic hazard health risk assessment.

Parameter	Physical Meaning	Unit	Children	Adults	Reference
*C*	Concentration of trace element in street dust	mg kg^−1^	95% UCL	95% UCL	This study
lngR	Ingestion rate	mg day^−1^	200	100	[27,28]
lnhR	Inhalation rate	m^3^ day^−1^	5	20	[23,28]
*EF*	Exposure frequency	day year^−1^	350	350	[28]
*ED*	Exposure duration	year	6	30	[27]
*SA*	Exposed skin area	cm^2^	1800	5000	[28]
*AF*	Adherence factor	mg cm^2^	1	1	[28]
*ABS*	Dermal absorption factor	-	0.03 for arsenic; 0.001 for other elements	0.03 for arsenic; 0.001 for other elements	[7,27,28]
*PEF*	Particle emission factor	m^3^ kg^−1^	1.32 × 10^9^	1.32 × 10^9^	[28]
*BW*	Average body weight	kg	15	55.9	[23,28]
*AT*	Averaging time	day	365*ED* (Non-carcinogens); 70 × 365 (Carcinogens)	365*ED* (Non-carcinogens); 70 × 365 (Carcinogens)	[7,27,28]

Note: 95% UCL indicates “the upper limit of the 95% confidence interval of the mean”.

**Table 2:** 95% UCL (mg kg^−1^) of heavy metal(loid)s in streets of Hezhang city and the RfD (mg kg^−1^ day^−1^) and SF (mg kg^−^^1^ day^−^^1^).

Parameter	Cr	Co	Ni	Cu	Zn	As	Cd	Sb	Pb	Hg
$C_{95\%UCL}$	162	19	44	230	1080	62	3.5	8.1	461	0.33
${\mathrm{RfD}}_{ing}$	3 × 10^−3^	2 × 10^−2^	2 × 10^−2^	4 × 10^−2^	0.3	3 × 10^−4^	0.001	4 × 10^−4^	3.5 × 10^−3^	3 × 10^−4^
${\mathrm{RfD}}_{inh}$	2.86 × 10^−5^	5.71 × 10^−6^	2.06 × 10^−2^	4.02 × 10^−2^	0.3	3 × 10^−4^	0.001	4 × 10^−4^	3.52 × 10^−3^	8.57 × 10^−5^
${\mathrm{RfD}}_{dermal}$	6 × 10^−5^	1.6 × 10^−2^	5.4 × 10^−3^	1.2 × 10^−2^	0.06	1.23 × 10^−4^	1 × 10^−5^	8 × 10^−6^	5.25 × 10^−4^	2.1 × 10^−5^
SF_ing_						1.50				
SF_dermal_						3.66				
SF_inh_	42	9.80	0.84			15.1	6.30			

**Table 3:** Descriptive statics of trace metal(loid)s, pH and OM in street dust of Hezhang city (*n* = 30) and soils and limestones in Guizhou province.

Parameters	Min	Max	AM	SD	Med	Skew	CV	LAS	RGS	PBG	CS	EF	LS
Cr	72	509	133	76	119	4.3	57	228	103	95.9	91.9	1.24	4
Co	11	30	18	4	18	1.0	21	43	41	19.2	10.5	0.94	16
Ni	27	68	41	9	39	1.4	23	86	76	39.1	28.1	1.00	89
Cu	48	1212	153	207	99	4.9	136	133	189	32.0	75.0	3.09	3
Zn	468	1723	966	307	1039	0.3	32	611	160	99.5	157.1	10.44	2
As	36	82	58	11	55	0.3	18	23	5	20.0	24.8	2.75	3
Cd	1.3	5.6	3.0	1.3	2.6	0.77	44	8.3	0.7	0.66	0.33	3.94	0.0
Sb	3.4	11.4	7.4	1.9	7.6	0.0	25	3.0	1.5	2.2	2.28	3.45	0.0
Pb	183	708	409	139	423	0.4	34	181	86	35.2	47.3	12.02	1
Hg	0.08	0.89	0.27	0.15	0.22	2.8	57	0.16	0.08	0.11	0.38	2.00	0.00
pH	7.3	9.7	8.5	0.6	8.5	−0.1	7	5.9	5.07	6.2	8.03	1.37	-
OM	1.9	9.6	5.0	1.5	4.7	1.1	31	3.70	3.53	4.26	4.97	1.10	-

Note: Units in mg kg^−1^ for trace metal(loid), % for OM, unitless for pH; Min, minimum; Max, Maximum; AM, arithmetical mean; Med, median; Skew, skewness; SD, arithmetical standard deviation; CV, coefficient of variation; LAS, mean of local agricultural soils (*n* = 23, this study); RGS: Regional grassland soil (*n* = 6); PBG, provincial background of surface soil in Guizhou [22]; CS, mean of control sites in Huaxi district of Guiyang; EF, enrichment factor = median/background of surface soil in Guizhou province; LS, mean of limestones in Guizhou (*n* = 3, this study); OM, organic matter.

**Table 4:** Comparison of trace metal(loid)s in street dust of different cities in the world (unit in mg kg^−1^, dry weight).

City	Cr	Co	Ni	Cu	Zn	As	Cd	Sb	Pb	Hg	Reference
Hezhang, China	119	18	39	99	1039	55	2.6	7.6	423	0.22	This study
Zhuzhou, China	115	13	35	98	1140	42	10.3	9.8	254	0.21	[9]
Huludao, China				162	1374		19.7		235	0.61	[7]
Avile’s, Spain	42	7	28	183	4892	17	22.3	8	514	2.56	[33]
Nanjing, China	126	11	56	123	394	13	1.1		103	0.12	[21]
Hangzhou, China	51	20	26	116	321		1.6		202	0.7	[37]
Guiyang, China	130		59	129	182	11	0.6		67	0.38	[38]
Xi’an, China	65			72	295	10		3.7	131	0.43	[39]
Baoji, China	123	123	42	113	612	18			383	1	[40]
Hongkong, China	324	10	42	534	4024		1.8		240	0.6	[41]
Beijing, China	86	11	45	138	723	24	2.3	12.3	168		[31]
Shanghai, China	264		66	258	753	8	1		237	0.14	[42]
Amman, Jordan	29	32	66	139	351		1.9		271		[43]
Kavala, Greece	196		58	124	272	17	0.2		301	0.10	[44]
Birmingham, UK			41	467	534		1.6		48		[45]
Luanda, Angola	26	3	10	42	317	5	1.2	3.4	351	0.13	[32]
Ottawa, Canada	42	8	15	38	101	1	0.3	0.4	33	0.02	[46]
Tehran, Iran	77		58	275	666	5	0.8	11	213		[47]
Shiraz, Iran	678		78	136	403	7	0.5	4.8	116	1.05	[30]
Changchun, China	96			68	465	23	0.6		94	0.24	[48]
Huainan, China	618	9		36		7	0.3	1.4	43	0.20	[34]
Suzhou, China	113			28	2258				45		[49]

**Table 5:** Correlation coefficient for metal(loid)s, pH, OM in street dust of Hezhang city (*n* = 30).

Element	Cr	Co	Ni	Cu	Zn	As	Cd	Sb	Pb	Hg	pH	OM
Cr	1											
Co	0.19	1										
Ni	0.49 **	0.70 **	1									
Cu	0.06	0.19	0.64 **	1								
Zn	0.10	0.15	0.37 *	0.54 *	1							
As	−0.15	−0.35	−0.34	0.04	0.21	1						
Cd	−0.15	0.14	0.04	0.12	0.38 *	0.01	1					
Sb	−0.13	−0.05	−0.04	0.30	0.57 **	0.80 **	0.27	1				
Pb	0.20	0.17	0.11	0.16	0.61 **	0.37 *	0.37 *	0.58 **	1			
Hg	−0.07	0.21	0.17	0.17	0.44 *	0.11	0.29	0.28	0.42 *	1		
pH	0.04	−0.15	−0.20	−0.27	−0.21	0.16	−0.13	−0.01	−0.10	0.40 *	1	
OM	−0.08	0.34	0.35	0.34	0.36	−0.11	0.13	0.08	0.28	0.62 **	0.50 **	1

Note: * Significant at 0.05 level (two tails); ** Significant at 0.01 level (two tails).

**Table 6:** Varimax rotated principal components analysis of metal(loid)s in Hezhang city (*n* = 30).

Component	PC-1	PC-2	PC-3
Cr	0.020	**0.653**	−0.354
Co	−0.331	**0.609**	0.381
Ni	−0.150	**0.948**	0.126
Cu	0.287	**0.642**	0.153
Zn	**0.544**	0.450	0.497
As	**0.887**	−0.261	−0.088
Cd	0.068	−0.057	**0.794**
Sb	**0.897**	0.026	0.256
Pb	**0.601**	0.244	0.446
Hg	0.189	0.123	**0.681**
Eigenvalues	3.3	2.4	1.2
% of Variance	32.6	24.2	12.0
Cumulative%	32.6	56.8	68.8

Note: Scores higher than 0.5 are shown in bold.

**Table 7:** Exposure dose, hazard quotients, and hazard index of studied metal(loid)s for children and adults in Hezhang city.

Element	$D_{ing}$	$D_{inh}$				$D_{dermal}$		$HQ_{ing}$		$HQ_{inh}$		$HQ_{dermal}$		*HI*	
	Child		Adult	Child	Adult	Child	Adult	Child	Adult	Child	Adult	Child	Adult	Child	Adult
Cr	2.07 × 10^−3^		2.78 × 10^−4^	3.92 × 10^−8^	4.21 × 10^−8^	1.86 × 10^−5^	1.39 × 10^−5^	6.90 × 10^−1^	9.26 × 10^−2^	1.37 × 10^−3^	1.47 × 10^−3^	3.11 × 10^−1^	2.32 × 10^−1^	**1.002**	0.326
Co	2.41 × 10^−4^		3.24 × 10^−5^	4.57 × 10^−9^	4.90 × 10^−9^	2.17 × 10^−6^	1.62 × 10^−6^	1.21 × 10^−2^	1.62 × 10^−3^	8.00 × 10^−4^	8.59 × 10^−4^	1.4 × 10^−4^	1 × 10^−4^	0.013	0.003
Ni	5.65 × 10^−4^		7.59 × 10^−5^	1.07 × 10^−8^	1.15 × 10^−8^	5.09 × 10^−6^	3.79 × 10^−6^	2.83 × 10^−2^	3.79 × 10^−3^	5.20 × 10^−7^	5.58 × 10^−7^	9.4 × 10^−4^	7 × 10^−4^	0.029	0.004
Cu	2.94 × 10^−3^		3.95 × 10^−4^	5.58 × 10^−8^	5.99 × 10^−8^	2.65 × 10^−5^	1.98 × 10^−5^	7.36 × 10^−2^	9.88 × 10^−3^	1.39 × 10^−6^	1.49 × 10^−6^	2.21 × 10^−3^	1.65 × 10^−3^	0.076	0.012
Zn	1.38 × 10^−2^		1.85 × 10^−3^	2.62 × 10^−7^	2.81 × 10^−7^	1.24 × 10^−4^	9.27 × 10^−5^	4.61 × 10^−2^	6.18 × 10^−3^	8.72 × 10^−7^	9.36 × 10^−7^	2.07 × 10^−3^	1.54 × 10^−3^	0.048	0.008
As	7.92 × 10^−4^		1.06 × 10^−4^	1.50 × 10^−8^	1.61 × 10^−8^	2.14 × 10^−4^	1.59 × 10^−4^	**2.64**	3.54 × 10^−1^	5.00 × 10^−5^	5.36 × 10^−5^	**1.74**	**1.30**	**4.379**	**1.650**
Cd	4.47 × 10^−5^		6.00 × 10^−6^	8.48 × 10^−10^	9.10 × 10^−10^	4.03 × 10^−7^	3 × 10^−7^	4.45 × 10^−2^	6 × 10^−3^	8.48 × 10^−7^	9.10 × 10^−7^	4.03 × 10^−2^	3 × 10^−2^	0.085	0.036
Sb	1.04 × 10^−4^		1.40 × 10^−5^	1.97 × 10^−9^	2.12 × 10^−9^	9.37 × 10^−7^	6.98 × 10^−7^	2.60 × 10^−1^	3.49 × 10^−2^	4.93 × 10^−6^	5.29 × 10^−6^	1.17 × 10^−1^	8.73 × 10^−2^	**0.377**	0.122
Pb	5.89 × 10^−3^		7.91 × 10^−4^	1.12 × 10^−7^	1.20 × 10^−7^	5.30 × 10^−5^	3.95 × 10^−5^	**1.68**	2.26 × 10^−1^	3.17 × 10^−5^	3.40 × 10^−5^	1.01 × 10^−1^	7.53 × 10^−2^	**1.785**	0.301
Hg	4.22 × 10^−6^		5.66 × 10^−7^	7.99 × 10^−11^	8.57 × 10^−11^	3.80 × 10^−8^	2.83 × 10^−8^	1.41 × 10^−2^	1.89 × 10^−3^	9.32 × 10^−7^	1.00 × 10^−6^	1.81 × 10^−3^	1.35 × 10^−3^	0.016	0.003

Note: Exposure dose, hazard quotients, and hazard index higher than 1 are shown in bold.

**Table 8:** Daily exposure dose and cancer risk of metal(loid)s in street dust to population in Hezhang city.

Parametes	Cr	Co	Ni	As	Cd
$LADD_{ing}$				1.13 × 10^−4^	
$LADD_{inh}$	2.14 × 10^−8^	2.49 × 10^−9^	5.84 × 10^−9^	8.19 × 10^−9^	4.63 × 10^−10^
$LADD_{dermal}$				8.66 × 10^−5^	
$Risk_{ing}$				**1.70 × 10^−4^**	
$Risk_{inh}$	**3.06 × 10^−6^**	4.35 × 10^−8^	2.19 × 10^−7^	4.46 × 10^−7^	1.82 × 10^−9^
$Risk_{dermal}$				**3.17 × 10^−4^**	
Cancer Risk	**3.06 × 10^−6^**	4.35 × 10^−9^	2.21 × 10^−7^	**4.90 × 10^−4^**	1.86 × 10^−10^

Note: Cancer risk of metal(loid)s higher than 1 × 10^−6^ are shown in bold.

**Table 9:** Comparisons of HIs for trace metal(loid)s in street dust of different cities.

Population Groups	City	Cr	Co	Ni	Cu	Zn	As	Cd	Sb	Pb	Hg	Reference
Children	Hezhang, China	**1.0**	1.3 × 10^−2^	2.9 × 10^−2^	7.6 × 10^−2^	4.8 × 10^−2^	**4.4**	8.5 × 10^−2^	**0.38**	**1.8**	1.6 × 10^−2^	This study
	Zhuzhou, China	**0.32**	6.3 × 10^−3^	1.5 × 10^−2^	2.9 × 10^−2^	8.1 × 10^−2^	**3.2**	**0.52**	**0.37**	**3.2**	**0.18**	[58]
	Huludao, China				4.8 × 10^−2^	**0.11**		**0.62**		**1.1**	**0.18**	[7]
	Beijing, China	**0.13**	3.9 × 10^−3^	1.7 × 10^−2^	2.8 × 10^−2^	1.8 × 10^−2^	**0.75**	2 × 10^−2^	**0.29**	**0.35**		[31]
	Nanjing, China	4.6 × 10^−3^	1.7 × 10^−3^	5.5 × 10^−3^	1.2 × 10^−2^	5.1 × 10^−3^		8.0 × 10^−3^		**0.13**		[21]
	Shiraz, Iran	1.2 × 10^−3^		4.9 × 10^−3^	8.8 × 10^−3^	6.4 × 10^−3^			1.4 × 10^−3^	**0.22**	**0.12**	[30]
	Luanda, Angola	6.6 × 10^−2^	1.1 × 10^−3^	3.6 × 10^−3^	7.3 × 10^−3^	7.4 × 10^−3^	**0.14**	1.0 × 10^−2^	6.6 × 10^−2^	**0.72**	1.6 × 10^−2^	[32]
Adult	Hezhang, China	**0.326**	3 × 10^−3^	4 × 10^−3^	1.2 × 10^−2^	8.0 × 10^−3^	**1.7**	3.6 × 10^−2^	**0.12**	**0.30**	3 × 10^−3^	This study
	Zhuzhou, China	4.5 × 10^−2^	1.2 × 10^−3^	2 × 10^−3^	4 × 10^−3^	1.1 × 10^−2^	**0.45**	7.4 × 10^−2^	5.1 × 10^−2^	**0.44**	**0.11**	[58]
	Huludao, China				5.7 × 10^−3^	1.4 × 10^−2^		**0.26**		**0.14**	8.8 × 10^−2^	[7]
	Beijing, China	1.7 × 10^−3^	6.9 × 10^−4^	2.2 × 10^−3^	3.6 × 10^−3^	2.3 × 10^−3^	**0.1**	2.5 × 10^−3^	3.9 × 10^−2^	4.5 × 10^−2^		[31]
	Nanjing, China	5.2 × 10^−3^	2.4 × 10^−4^	6.0 × 10^−4^	1.3 × 10^−3^	5.5 × 10^−4^		9.3 × 10^−4^		1.4 × 10^−2^		[21]
	Shiraz, Iran	2 × 10^−4^		7.2 × 10^−4^	9.0 × 10^−4^	7.8 × 10^−4^			1.1 × 10^−4^	4.9 × 10^−2^	6.1 × 10^−2^	[30]

Note: HIs for trace metal(loid)s higher than 0.1 are shown in bold.

**Table 10:** Comparisons of cancer risk in street dust of different cities.

City	As	Cr	Ni	Cd	Co	Reference
Hezhang, China	4.9 × 10^−4^	3.1 × 10^−6^	2.2 × 10^−7^	1.8 × 10^−9^	4.6 × 10^−8^	This study
Zhuzhou, China	1.1 × 10^−6^	3.2 × 10^−8^	6.9 × 10^−9^	8.5 × 10^−8^	3.2 × 10^−8^	[58]
Huludao, China				2.4 × 10^−8^		[7]
Beijing, China	2.4 × 10^−8^	2.0 × 10^−7^	2.1 × 10^−9^	9.5 × 10^−10^	5.6 × 10^−9^	[31]
Nanjing, China		3.9 × 10^−8^	7.2 × 10^−10^	3.1 × 10^−10^	2.3 × 10^−9^	[21]
Shiraz, Iran		1.1 × 10^−8^	8.3 × 10^−8^			[30]
Luanda, Angola	7.7 × 10^−6^	5.7 × 10^−8^	4.6 × 10^−10^	3.9 × 10^−10^	1.5 × 10^−9^	[32]
